# Early Growth Response Protein 1 Exacerbates Murine Inflammatory Bowel Disease by Transcriptional Activation of Matrix Metalloproteinase 12

**DOI:** 10.3390/biomedicines12040780

**Published:** 2024-04-02

**Authors:** Shih-Yao Chen, Chuan-Yin Fang, Bing-Hwa Su, Hao-Ming Chen, Shih-Chi Huang, Po-Ting Wu, Ai-Li Shiau, Chao-Liang Wu

**Affiliations:** 1 Department of Nursing, College of Nursing, Chung Hwa University of Medical Technology, Tainan 717302, Taiwan; leonlai50@gmail.com; 2 Division of Colon and Rectal Surgery, Ditmanson Medical Foundation Chia-Yi Christian Hospital, Chiayi 600566, Taiwan; 3 School of Respiratory Therapy, College of Medicine, Taipei Medical University, Taipei 110301, Taiwan; 4 Department of Biochemistry and Molecular Biology, College of Medicine, National Cheng Kung University, Tainan 701401, Taiwan; 5 Department of Orthopedics, College of Medicine, National Cheng Kung University, 1 University Road, Tainan 701401, Taiwan; 6 Department of Orthopaedics, National Cheng Kung University Hospital, College of Medicine, National Cheng Kung University, Tainan 701401, Taiwan; 7 Medical Device Innovation Center, National Cheng Kung University, Tainan 701401, Taiwan; 8 Department of Biomedical Engineering, National Cheng Kung University, Tainan 701401, Taiwan; 9 Department of Microbiology and Immunology, College of Medicine, National Cheng Kung University, Tainan 701401, Taiwan; 10 Department of Medical Research, Ditmanson Medical Foundation Chia-Yi Christian Hospital, Chiayi 600566, Taiwan

**Keywords:** inflammatory bowel disease, early growth response protein 1, dextran sulfate sodium, metalloproteinase 12

## Abstract

Inflammatory bowel disease (IBD) is an inflammatory condition affecting the colon and small intestine, with Crohn’s disease and ulcerative colitis being the major types. Individuals with long-term IBD are at an increased risk of developing colorectal cancer. Early growth response protein 1 (Egr1) is a nuclear protein that functions as a transcriptional regulator. Egr1 is known to control the expression of numerous genes and play a role in cell growth, proliferation, and differentiation. While IBD has been associated with severe inflammation, the precise mechanisms underlying its pathogenesis remain unclear. This study aimed to investigate the role of Egr1 in the development of IBD. High levels of Egr1 expression were observed in a mouse model of colitis induced by dextran sulfate sodium (DSS), as determined by immunohistochemical (IHC) staining. Chronic DSS treatment showed that *Egr1* knockout (KO) mice exhibited resistance to the development of IBD, as determined by changes in their body weight and disease scores. Additionally, enzyme-linked immunosorbent assay (ELISA) and IHC staining demonstrated decreased expression levels of proinflammatory cytokines such as IL-1β, IL-6, and TNF-α, as well as matrix metalloproteinase 12 (MMP12). Putative Egr1 binding sites were identified within the MMP12 promoter region. Through reporter assays and chromatin immunoprecipitation (ChIP) analysis, it was shown that Egr1 binds to the MMP12 promoter and regulates MMP12 expression. In conclusion, we found that Egr1 plays a role in the inflammation process of IBD through transcriptionally activating MMP12.

## 1. Introduction

Inflammatory bowel disease (IBD) is a broad term encompassing inflammatory conditions affecting the intestines. It involves chronic inflammation in both the large and small intestine, impacting the entire digestive tract and colon mucosa. In clinical classification, it is commonly divided into two categories based on the site of onset and degree of invasion: Crohn’s disease and ulcerative colitis [1]. The cause of IBD remains unclear, with various factors implicated, including the patient’s genetic susceptibility, intestinal microorganisms, environmental influences, immune system imbalances, hygiene, and diet. The interactive regulation of these multiple factors can contribute to the onset of IBD [2]. Chronic inflammation in the intestines, a hallmark of IBD, is also associated with an increased risk of colorectal cancer [3].

Egr1 (Early growth response protein 1), also known as Zif268 and NGFI-A, is a member of the early growth response protein (EGR) gene family. This zinc finger transcriptional regulator binds to GC-rich regions of DNA, thus modulating the expression of numerous genes involved in cell differentiation, growth, and apoptosis [4]. In the context of tumor cells, Egr1 plays a dual role, functioning as both a tumor suppressor gene and a tumor-promoting gene [5]. Egr1 also plays a crucial role in promoting proinflammatory cytokines during inflammation and has the ability to activate the production of downstream inflammatory genes [6]. At present, the relationship between IBD and Egr1 is not entirely clear. However, it has been observed that, in specimens from patients with inflammation, whether from Crohn’s disease or ulcerative colitis, the expression level of Egr1 is higher compared to that of normal tissues [7]. During the inflammatory process, the expression of proinflammatory mediators, such as interleukin-1β (IL)-1β and tumor necrosis factor-α (TNF)-α, can regulate an increase in the expression of Egr1 [8]. Elevated levels of Egr1, in turn, negatively feedback to these proinflammatory substances, potentially intensifying inflammation [8]. This highlights the significant role of Egr1 in the context of IBD.

Matrix metalloproteinases (MMPs) are endogenous peptidases dependent on zinc and calcium ions. Initially synthesized as inactive zymogens, MMPs require self-cleavage and association with zinc ions to become proteolytically active. Their primary function is to degrade proteins within the extracellular matrix (ECM). To date, researchers have identified at least 28 different types of MMPs, named based on their ability to cleave various components of the ECM [9]. MMPs play a vital role in normal physiological functions. Their ability to remodel the ECM contributes to embryo development, cell movement, proliferation, apoptosis, and blood vessel formation, all of which necessitate appropriate MMP levels [10,11]. Conversely, excessive MMP expression can lead to irreversible degradation, contributing to various diseases, including tumor progression and metastasis [12], inflammatory responses [13], and rheumatoid arthritis [14]. Previous studies have identified the overexpression of MMPs in clinical patients with IBD and speculated that MMPs can inflict damage on the intestinal mucosal tissue [15,16] and potentially impede the speed of tissue repair [17]. Consequently, it has been established that an excess of MMPs may contribute to harm to the intestinal mucosa, potentially influencing the progression of IBD.

There are various methods for inducing signs in IBD animal models, such as adding dextran sodium sulfate (DSS) to drinking water or administering TNBS/Oxazolone. These two drugs influence the intestinal inflammatory response, cytokine secretion, and cell function, commonly employed in research on attachment and immunotherapy. Conversely, mice with partial gene knockouts, like multidrug resistance protein 1 (MDR1) or inhibitor of nuclear factor kappa-B kinase subunit alpha (IKKα), are also utilized in IBD mouse model research. The DSS drug used in this study initially increases the permeability of the intestinal mucosa after oral administration, leading to an upsurge in the expression of proinflammatory factors. Subsequently, the barrier function of intestinal epithelial cells is compromised, giving rise to a significant influx of inflammatory cells. Additionally, infiltrating microorganisms entering the intestine can trigger an immune response, creating an inflammatory state that mimics the symptoms of IBD [18].

Currently, it is known that Egr1 is highly expressed in clinical patient specimens. However, the functional role of Egr1 in this context has been scarcely investigated. Therefore, this study aims to utilize an IBD mouse model induced by DSS to elucidate the molecular mechanisms responsive to EGR1. *Egr1* gene knockout (KO) mice are employed to demonstrate the crucial role of Egr1 in the development of IBD. Subsequently, cell experiments are conducted to examine whether the expression of the *Egr1* gene influences the expression levels of MMPs and to elucidate the regulatory mechanisms affecting MMP expression and inflammatory cytokines. This exploration helps to understand how these factors contribute to the onset of IBD.

## 2. Materials and Methods

### 2.1. DSS-Induced IBD Model

Nine-week-old *Egr1*^−/−^ C57BL/6 mice were purchased from The Jackson Lab (600 Main Street, Bar Harbor, ME, USA) and housed in the Laboratory Animal Center of the National Cheng Kung University. The mice were maintained in specific pathogen-free (SPF) animal care facilities under isothermal conditions with regular photoperiods. The experimental protocol adhered to the rules of the Animal Protection Act of Taiwan and was approved by the Laboratory Animal Care and Use Committee of the National Cheng Kung University (109106). DSS [M.W. 36,000–50,000 (MP Biomedicals, LLC, Eschwege, Germany)] was added to drinking water at a concentration of 0.5% for each C57BL/6 mouse. Fresh DSS solution was prepared daily. The mice were exposed to 0.5% DSS for 30 days. Healthy control animals received water only. The clinical signs recorded in the colitis mice included survival, body weight, stool consistency, fecal bleeding, and diarrhea, as described previously [19].

### 2.2. Histological Observations and Scoring

Colons were removed from the mice and assessed for diarrhea and fecal blood by a pathophysiologist who was blinded to the experiment. Subsequently, the colon specimens were fixed in 10% paraformaldehyde, embedded in paraffin, and sliced into sections. These sections were stained with hematoxylin and eosin (H&E), and histological analysis was performed in a blinded fashion. Diarrhea score was evaluated as previously described [19].

### 2.3. Immunohistochemistry (IHC)

Colons were removed from the mice and rinsed with chilled phosphate-buffered saline (PBS). The tissues were embedded in OCT compound, and serial transverse sections of the colon, each 5 μm thick, were fixed in 4% formaldehyde. After being washed twice with PBS, the colon sections were incubated in cold acetone for 1 min. Before incubation with the primary antibodies, the colon sections underwent 5 min of incubation in 3% H_2_O_2_ to block their endogenous peroxidase activity. Monoclonal antibodies, anti-Egr1 (SantaCurz Biotechnology, Dallas, TX, USA), and anti-MMP12 (SantaCurz) were used to identify protein expression. Following overnight incubation at 4 °C, the slides were rinsed twice in PBS, and then the secondary antibodies (Jackson ImmunoResearch Laboratories, West Grove, PA, USA) were applied. AEC substrate solution (Zymed Laboratory, South San Francisco, CA, USA) was used as a substrate. The tissue sections were washed in PBS twice and counterstained with hematoxylin, as described previously [19].

### 2.4. Enzyme-Linked Immunosorbent Assay (ELISA) for Cytokine Detection in the Colonic Tissue

Colons were removed from the mice and rinsed with chilled PBS. After assessing the specimens for diarrhea and fecal blood, they were placed in PBS supplemented with protease inhibitor cocktails. Following homogenization, the samples underwent centrifugation at 16,000× *g* for 10 min at 4 °C to precipitate insoluble cellular debris, and the supernatant was stored at −80 °C until analysis. The concentrations of TNF-α, IL-6, and IL-1β will be evaluated using mouse ELISA kits (R&D Systems, Minneapolis, MN, USA), as described previously [19,20].

### 2.5. Cell Lines

A human colorectal carcinoma cell line, HCT-116 cells, was cultured in McCoy’s 5A culture medium (HyClone, Logan, UT, USA) supplemented with 10% fetal bovine serum (FBS) (HyClone) and 50 μg/mL of gentamicin in a 5% CO_2_ environment at 37 °C. Human embryonic kidney (HEK) 293T cells were cultured in Dulbecco’s Modified Eagle’s Medium (DMEM) containing 10% Cosmic Calf Serum (CCS) (HyClone) and 50 μg/mL of gentamicin. The culture was maintained in a 37 °C incubator with 5% CO_2_.

### 2.6. Production of Lentiviral Vectors

Initially, 4 × 10^6^ HEK 293T cells were distributed evenly onto a 10 cm culture dish. On the following day, the culture medium was replaced with 8 mL of DMEM containing 10% Cosmic Calf Serum (CCS). After 1 h, transfection was performed using pSPAX2, pMD2.G, pLKO.1-shRNAs [pLKO.1-shLuc(TRCN0000072246), pLKO.1-shEgr1#1(TRCN000013833), and pLKO.1-shEgr1#2(TRCN000013834)] were purchased from the Biomedical Translation Research Center, Academia Sinica, Taipei, Taiwan), and 250 μL of HEPES-containing water (125 μL 1 M HEPES, pH 7.4, dissolved in 50 mL Mini.Q water). A total of 250 μL 0.5 M CaCl_2_ was added and mixed thoroughly. Finally, 500 μL of 2× HeBS (0.05 M HEPES, 0.28 M NaCl, 1.5 mM Na_2_HPO_4_, pH 7.0) was slowly added, incubated for 20 min, then a total volume of 1000 μL was evenly added to the culture dish. After 16 h, this was replaced with DMEM containing 2% CCS, the virus-containing culture medium was collected after 48 h, and stored at −80 °C, as described before [21,22,23].

### 2.7. Lentiviral Infection and Stable Transfectant Selection

HCT-116 cells were placed in a 6-well plate. On the next day, the culture medium was replaced with the virus liquid. In total, 1 mL of virus liquid (containing 8 μg polybrene) was added to each well for cell infection. After 8 h of infection, this was replaced with 10% FBS McCoy’s 5A medium and cultured for an additional 40 h. Subsequently, the culture medium was changed to one containing 2 μg/mL of puromycin and continued culturing for one week to screen out the infected cells. Finally, the culture medium was replaced with normal culture medium, as described previously [21,22].

### 2.8. Cloning for Human MMP12 Promoter

Utilizing chromosomal DNA extracted from A549 cells as a template, a polymerase chain reaction (PCR) was performed using h.MMP-12 promoter primers (Forward: 5′- GGCGAATTCCAAGCTTAAAGTAGC-3′; Reverse: 5′-CAGATCTTACTGTGTTC CTTTCTAGCCTA-3′). The PCR conditions involved an initial denaturation at 94 °C for 5 min, followed by 40 cycles of denaturation at 94 °C for 1 min, annealing at 56 °C for 3 min and 30 s, extension at 72 °C for 3 min and 30 s, and a final extension at 72 °C for 7 min. The reaction was terminated at 4 °C indefinitely, and the expected product size was 3030 bp. Subsequently, the PCR product was connected to the TA vector (Yeastern Biotech, Taipei City, Taiwan) for sequencing. The sequenced product was then subcloned into the pFRL2 plasmid using *Sma*I + *BamH*I restriction enzymes. After successful subcloning, the constructed plasmid was transfected into HEK 293T cells to evaluate the promoter’s activity.

### 2.9. Cell Transfection

Then, 4 × 10^4^ HCT116 cells were cultured in 24-well plates, and 1 μg of plasmid was delivered into the cells using polyethylenimine (PEI) (25 k, 0.45 mg/mL, pH 7.0). DNA (μg) and PEI (μL) were mixed at a ratio of 1:3 for 5 min, and then combined with culture medium containing 2% FBS. After a 20 min incubation at room temperature, the mixture was added to the cell culture. After 6 h, it was replaced with serum culture medium containing 2% FBS.

### 2.10. Reporter Gene Assay

HCT116 cells were cultured in a 24-well plate and transfected with pFRL2 and pFRL2-h.MMP12 promoter plasmids. After 48 h of transfection, the cells were washed with PBS, 150 μL dissociation buffer (containing 0.01 M DTT) was added, and placed on ice for 20 min. Centrifugation at 12,000× *g* was performed for 10 min at 4 °C, 40 μL of the sample was taken, and 20 μL Luciferase Assay Reagent II (Dual-Luciferase^®^ Reporter 1000 Assay System, Promega, Madison, WI, USA) was added to measure firefly luciferase activity as the target gene expression quantity. After the reaction, 20 μL of Stop & Glo^®^ Reagent (containing Substrate, 50×) (Dual-Luciferase^®^ Reporter 1000 Assay System, Promega) was added to measure the expression of the CMV promoter-driven renilla luciferase activity as an internal control group.

### 2.11. Chromatin Immunoprecipitation (ChIP) Assay

Then, 1 × 10^7^ Hct166 cells were plated in a 10 cm culture dish containing 7 mL of 10% FBS DMEM. The next day, fresh formalin was added directly to a final concentration of 1% and incubated for 10 min to enhance crosslinking. A total of 0.125 M glycine was added to stop crosslinking, and then the culture medium was removed and 20 mL of PBS was added with added protease inhibitors. The cells were washed twice, scraped, and centrifuged at 900× *g* for 5 min. After removing the supernatant, 1 mL of SDS lysis buffer was added with protease inhibitors and placed on ice for 10 min. A sonicator was used to shear the chromosomal DNA into 500 to 1000 bp fragments on ice, and this was confirmed with a 1% agarose gel. Centrifugation at 13,000× *g* was performed for 10 min to remove the pellet, and 100 μL of the supernatant was diluted 10 times in ChIP dilution buffer. In total, 60 μL of Protein G agarose was added and incubated at 4 °C for 1 h to avoid nonspecific binding. The Protein G agarose was removed, then the supernatant was placed into the ChIP dilution buffer. Anti-RNA polymerase II (positive control), mouse IgG (negative control), or anti-Egr1 primary antibody was added to the supernatant and incubated overnight at 4 °C. The next day, 60 μL of protein G agarose was added, incubated at 4 °C for 1 h, centrifuged at 3500× *g* for 1 min, the supernatant was removed, and washed with different salt gradient buffers (Low Salt Immune Complex Wash Buffer, High Salt Immune Complex Wash Buffer, LiCl Immune Complex Wash Buffer) and TE buffer several times. Finally, as much remaining buffer was absorbed as possible, 100 μL of elution buffer was added, shaken gently at room temperature for 15 min, and then the supernatant was absorbed to a new volume. This was repeated twice with a 1.5 mL Eppendorf tube. A total of 8 μL of 5 M NaCl was added to the supernatant and incubated at 65 °C overnight. Then, 1 μL of RNase A was added at 37 °C for 30 min, followed by 4 μL of 0.5 M EDTA, 8 μL of 1 M Tris-HCl (pH 6.5), and 1 μL of 10 mg/mL proteinase K. After incubation at 45 °C for 2 h, the DNA was purified and analyzed by polymerase chain reaction and 2% agarose gel electrophoresis (EZ-ChIP™, Merck Millipore, Burlington, MA, USA), as described previously [24].

### 2.12. Statistics

Data are expressed as the mean ± SEM. Differences between two groups and among groups were analyzed using Student’s *t* test and one-way ANOVA followed by Dunnet multiple comparison tests, respectively. Differences in body weight changes were compared by repeated-measures analysis of variance. The percent survival was compared by the log-rank (Mantel-Cox) test (Prism 5.0). *p* values less than 0.05 are considered as significant.

## 3. Results

### 3.1. Egr1 Is Highly Expressed in Colon Epithelial Cells of IBD Mice

**Figure 1:**
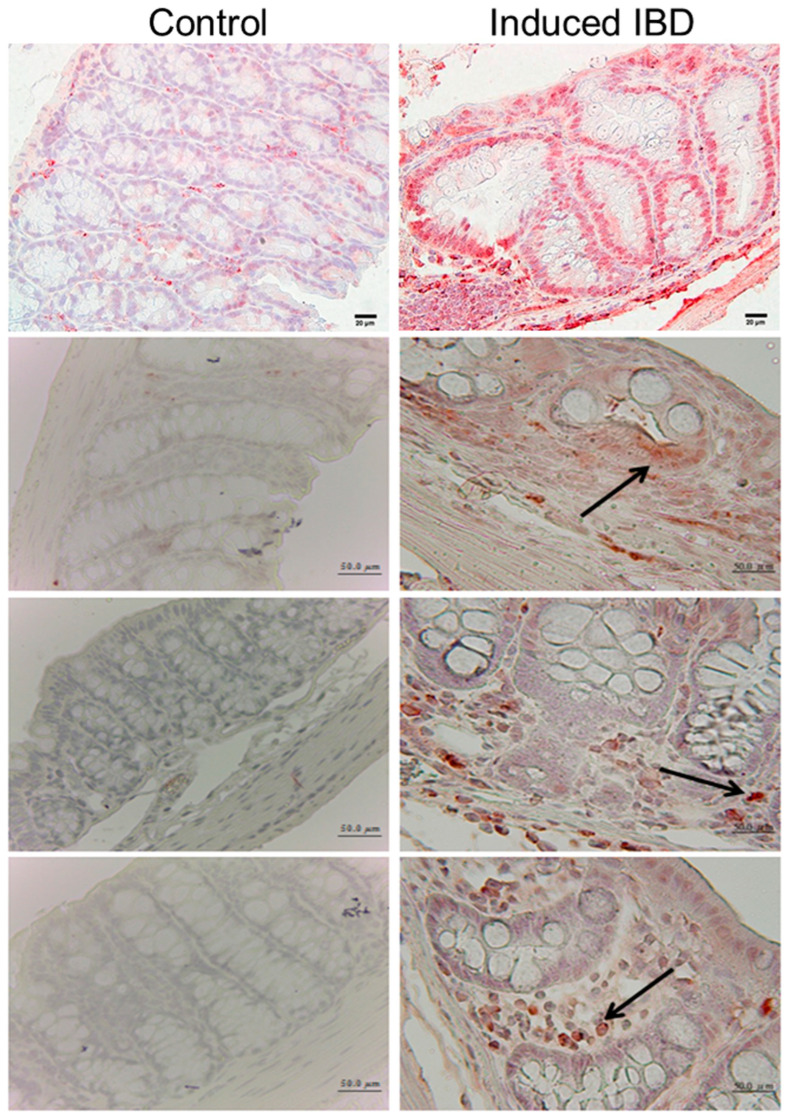
Expression of early growth response protein 1 (Egr1) in the intestine of inflammatory bowel disease (IBD) mice. C57BL/6 wild-type mice were given access to regular drinking water (Control) and 0.5% DSS, respectively (Induced IBD). The large intestines of successfully induced IBD mice and the large intestines of normal mice were then removed, fixed in formalin, embedded in paraffin, and sectioned. Immunohistochemical (IHC) staining was performed to analyze the protein expression of Egr1. The arrow indicates the location of Egr1 within the cells. Scale bars represent 20 and 50 μm in ×40 and ×100 magnifications, respectively.

According to the previous literature, it was found that, in patients with IBD, the expression level of Egr1 was significantly higher in tissue with Crohn’s disease or ulcerative colitis than in adjacent normal tissue [7]. Therefore, it is speculated that the overexpression of Egr1 may contribute to IBD, being one of the causes of the illness. To investigate this further, we employed the mouse IBD model induced by DSS. Immunohistochemical staining was utilized to detect the expression of the Egr1 protein. It was observed that Egr1 was overexpressed in the intestines of mice with successfully induced IBD (Figure 1).

### 3.2. Clinical Signs of DSS-Induced Egr1 Gene KO and Wild-Type Mice

**Figure 2:**
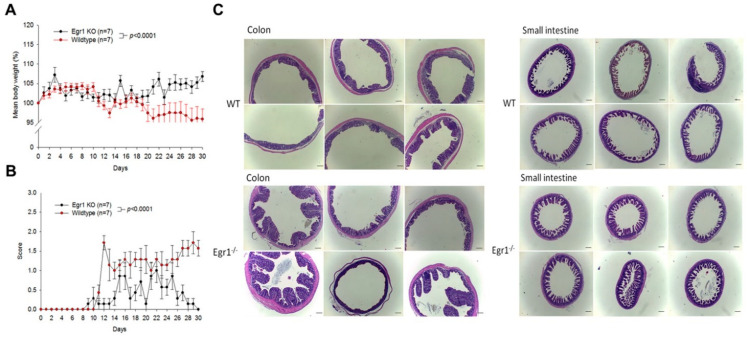
Mean body weight, disease score, and colon and small intestine sections in DSS-induced *Egr1* knockout (KO) mice. Nine-week-old C57/BL6 wild-type (WT) and *Egr1* homozygous KO (*Egr1*^−/−^ mice) were orally administrated with water containing 0.5% DSS for 30 days. (**A**) Mean body weight, (**B**) disease score, and (**C**) colon and small intestine were demonstrated by H&E staining. Scale bars represent 20 μm in ×40 magnification.

Although we identified elevated Egr1 expression in the IBD mice, it was not conclusively proven whether increased Egr1 expression contributes to a higher likelihood of IBD occurrence. Therefore, in the DSS-induced mouse IBD model, a comparison was made between *Egr1* gene KO (*Egr1*^−/−^) and wild-type (WT) mice. Figure 2A reveals a significant difference in the body weight changes between the two groups when exposed to 0.5% DSS. Wild-type mice displayed a more pronounced weight loss trend, indicating a greater sensitivity to DSS. Regarding diarrhea scores, wild-type mice exhibited more severe diarrhea than *Egr1* KO mice, with the latter showing a gradual reduction in signs during the later stages (Figure 2B). This suggests that *Egr1* gene KO mice demonstrate enhanced tolerance following DSS exposure.

An examination of intestinal tissue sections revealed that wild-type mice drinking DSS displayed significantly thicker and shorter small intestinal villi, along with damaged and structurally incomplete colonic mucosa. In contrast, *Egr1* gene KO mice drinking DSS exhibited a more complete structure in both the small intestine and colon, with no evident damage (Figure 2C). This observation suggests that mice lacking Egr1 expression possess superior protective capabilities, maintaining their intestinal integrity and mitigating inflammation, thereby reducing the likelihood of symptom deterioration.

### 3.3. Reduced Expression Levels of MMPs and Proinflammatory Cytokines in the Intestines of Egr1 KO Colitis Mice

**Figure 3:**
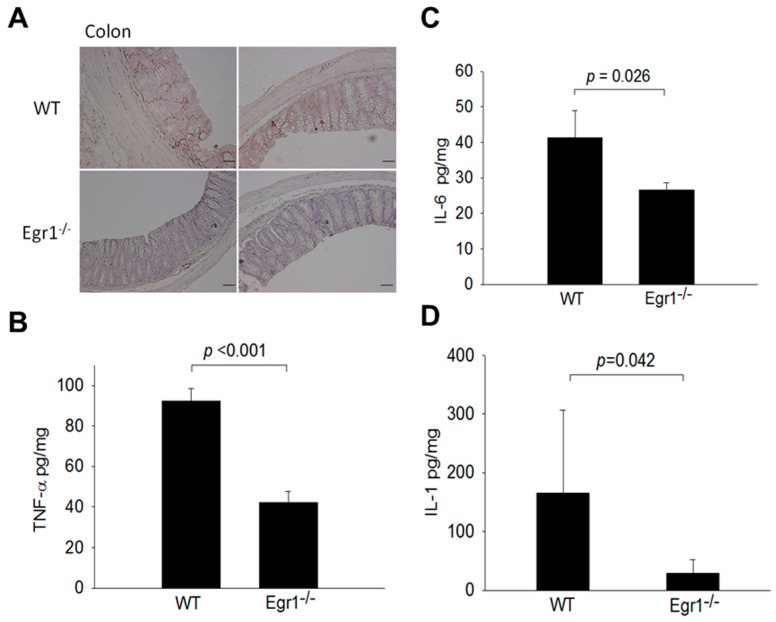
Expression levels of matrix metalloproteinase 12 (MMP12) and proinflammatory cytokines in DSS-induced *EGR1* KO mice. Nine-week-old C57/BL6 wild-type (WT) and *Egr1* KO (Egr1^−/−^ mice) were orally administrated with water containing 0.5% DSS for 30 days. (**A**) MMP-12, (**B**) TNF-α, (**C**) IL-6, and (**D**) IL-1 expression in colon tissue section and extracts, as determined by IHC and ELISA. Scale bars represent 20 μm in ×40 magnification. Values are the mean ± SEM.

According to previous studies, MMP12 is noted for its overexpression in the colons of patients with IBD [25]. To investigate the potential association between Egr1 and the expression of MMPs, IHC staining was initially employed to assess the levels of MMP12 expression. The results revealed that the expression levels of MMP12 in the intestines of *Egr1* knockout mice were significantly lower than those in wild-type mice (Figure 3A). Furthermore, ELISA was utilized to measure the expression levels of proinflammatory cytokines, including IL-1, IL-6, and TNF-α. The results demonstrated that, in *Egr1* knockout mice, the expression levels of TNF-α, IL-6, and IL-1 were lower compared to those in wild-type mice (Figure 3B–D).

### 3.4. Egr1 Directly Binds to the MMP12 Promoter and Increases MMP12 Promoter Activity

**Figure 4:**
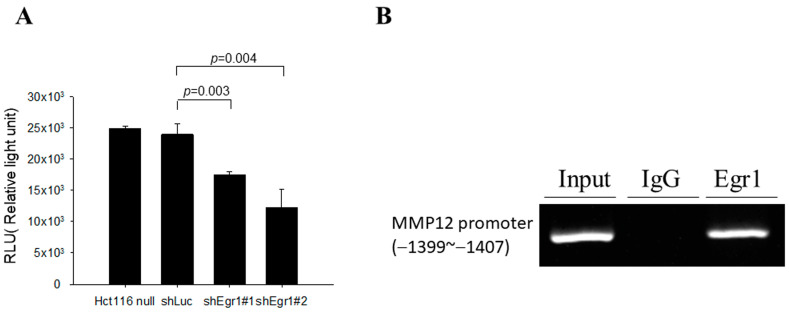
Egr1 directly binds to the MMP-12 promoter. (**A**) Hct116 cells transduced with LVShEgr1 and LVshLuc were transfected with pFRL2-h.MMP12 promoter. Total cell lysates were collected 48 h post-transfection and luciferase activities were determined using a chemiluminescence analyzer. (**B**) Chromatin immunoprecipitation (ChIP) assay was performed in Hct166 cells using the anti-Egr1 antibody, with normal IgG serving as the negative control.

Egr1 is known to be a common transcription factor involved in the regulation of many genes, affecting downstream expression and phenotypes. To further confirm whether and how Egr1 regulates MMP12 gene expression, a luciferase reporter assay was initially employed to assess the impact of inhibiting Egr1 expression on MMP12 promoter activity. HCT116 cells were first infected with lentiviruses carrying shLuc, shEgr1, or a control without any genes. Subsequently, cell lines with stable expression were screened using puromycin, and the pFRL2-h.MMP12 promoter was introduced through transfection. After 48 h, protein was harvested to measure the luciferase activity. The results demonstrated that, as the expression of the Egr1 gene decreased, the activity of the MMP12 promoter also decreased (Figure 4A). Next, through the analysis of the bioinformatics software Vector NTI Advance (TM) 11.0 (Invitrogen, Carlsbad, CA, USA), it was found that the MMP12 promoter from positions −1399 to −1407 contains the Egr1 binding sequence ‘GCG(T/C)GGGCG’, as reported by Christy and Nathans [26], with a similarity of 77.8%. Through a ChIP assay, Egr1 directly binds to the MMP12 promoter region between −1399 and −1407 (Figure 4B).

### 3.5. Egr1 Induces MMP12 Expression at the Transcriptional Level

**Figure 5:**
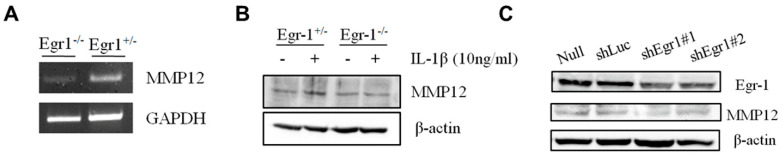
Expression of MMP12 in *Egr1*-deficient cells and mouse colon. (**A**) MMP12 expression in the colon from *Egr1* heterozygous (*Egr1*^+/−^) and homozygous KO (*Egr1*^−/−^) mice, as determined by RT-PCR. (**B**) MMP12 expression in murine embryonic fibroblasts (MEFs) from *Egr1*^+/−^ and *Egr1*^−/−^ mice stimulated with or without IL-1β (10 ng/mL) for 24 h, as determined by immunoblotting. (**C**) Hct116 cells were transduced with a lentiviral vector expressing short-hairpin RNA targeting Egr1 (shEgr1) and luciferase (shLuc, the control vector). Stable transfectants were then subjected to immunoblotting to examine Egr1 and MMP12 expression.

From the above, it is evident that Egr1 can bind to the promoter of MMP12, regulating the expression of the MMP12 gene. To delve deeper into this regulation at the transcriptional level, we extracted the RNA from the large intestines of both *Egr1* heterozygous (*Egr1*^+/−^) and *Egr1* homozygous KO (*Egr1*^−/−^) mice. RT-PCR was employed to analyze the expression of MMP12 mRNA. Figure 5A reveals that, following DSS induction, *Egr1*^−/−^ mice exhibited a lower MMP12 mRNA expression compared to *Egr1*^+/−^ mice. Thus, it becomes apparent that Egr1 indeed promotes MMP12 gene expression at the transcriptional level. Next, mouse embryonic fibroblasts (MEFs) were treated with IL-1β (10 ng/mL) to simulate an inflammatory condition. After 24 h, proteins were collected and MMP12 protein expression was analyzed through immunoblotting. The results demonstrated that the protein level of MMP12 in *Egr1*^−/−^ mice was lower than that in *Egr1*^+/−^ mice (Figure 5B). Furthermore, the analysis extended to HCT116 cells infected with lentivirus-expressing shEgr1 and they were screened for stable expression. The findings mirrored those in MEF cells—HCT116 cells with a suppressed Egr1 gene exhibited a significantly lower protein expression of MMP12 (Figure 5C). This collective evidence solidifies Egr1 as an upstream regulator of MMP12, affirming its capability to influence MMP12 expression at the transcriptional level.

## 4. Discussion

Egr1 was initially identified as an early response gene in cells, displaying a rapid responsiveness to various stimuli during growth [27]. Furthermore, it also plays a crucial role in cell proliferation and differentiation [28]. Previous studies have indicated elevated expression levels of certain chemokines and proinflammatory cytokines in IBD patients, and additionally, Egr1 expression in microarray results exhibits a positive correlation [13]. This study aimed to explore whether the excessive expression of Egr1 participates in the inflammatory response and how it regulates the expression of the downstream gene MMP12, leading to the formation of IBD. In our results, the expression of cytokine secretion related to the inflammatory response in the large intestine of *Egr1* KO mice was significantly lower than that in wild-type mice induced by DSS. Moreover, the IBD scores and weight loss were milder in the KO mice than in the wild-type mice. We speculate that Egr1 plays a regulatory role in the inflammatory response, and a high expression level of Egr1 may exacerbate IBD. The relationship between Egr1 and inflammation-related factors is also evident in other diseases. In the inflammatory response caused by ischemic tissue damage, Egr1 regulates the expression of IL-1β [29]. In addition, the treatment of lung epithelial cells with cigarette extract results in the regulation of IL-1β and TNF-α by Egr1, leading to increased expression [30]. In the context of intestinal endothelial cell injury, the activation of the ERK pathway occurs, subsequently increasing the expression of Egr1. Egr1, in turn, regulates downstream genes associated with inflammation and angiogenesis [31]. Egr1 appears to function in the regulation of proinflammatory substance expression, intensifying the inflammatory response. However, conflicting studies suggest that IL-1β secretion in the inflammatory response can elevate Egr1 expression [32]. Another study proposes that, in the JNK-MKK7-c-Jun pathway, IL-1β triggers Egr1 expression through c-Jun binding to the AP-1 position of the Egr1 promoter. The subsequent increase in Egr1 expression then influences the expression of downstream genes [33]. The expression of Egr1 is also regulated by miRNA. Egr1 may be the target gene of mmu-miR-7578, which can inhibit the expression of Egr1 mRNA by binding to the 3′-UTR of a specific sequence. Furthermore, IL-1β and TNF-α are also affected by Egr1, leading to decreased expression [34]. Therefore, we speculate that, when cells are damaged, a high expression of Egr1 will be induced, further regulating the expression of inflammatory factors, causing an inflammatory response. Simultaneously, the high expression of these inflammatory factors will also promote the expression of Egr1, creating a mutual regulation that produces a synergistic effect, leading to severe inflammation and the formation of IBD.

Previous studies have highlighted the significant role of MMPs in IBD, influencing the migration of inflammatory cells, ulceration of the intestinal mucosa, and matrix cleavage. Within the intestine, certain MMPs are consistently and stably expressed to maintain environmental balance, while others are upregulated in response to inflammation during tissue damage [35]. The behavior of MMPs in the intestine plays a crucial role in shaping the overall environment. As the damaged intestine initiates the repair process, intestinal epithelial cells gradually migrate to the ulcer site. During this migration, they induce the expression of MMP12 and MMP14, attracting immune cells and myofibroblasts. Additionally, various cell types, including myofibroblasts and stromal cells, express MMP1, MMP3, MMP9, MMP12, and MMP14 to facilitate matrix breakdown. This breakdown allows cells to enter the damaged area, initiating the repair process [36]. In this study, the expression level of MMP12 in *Egr1* KO mice after DSS induction was lower than that in the wild-type mice. Some unpublished data in our laboratory also revealed that the excessive expression of prothymosin-α can induce IBD by regulating the expression of MMP7 and MMP12. From the above, it can be inferred that the expression of MMP12 plays an important role in IBD. In terms of gene regulation, the binding sequence of Egr1 is repeated with the binding sequence of the transcription factor SP1, but Egr1 has a high binding competitiveness and can promote or inhibit the expression of downstream genes [30]. If the promoter of the inflammatory genes, TNF-α, IL1β, or IL6, has an SP1 binding site or an Egr1 binding site, Egr1 can bind to it and promote its expression. This study first discovered that Egr1 can regulate the expression of MMP12 by binding to the promoter of MMP12. The same situation applies to transcription and translation. Egr1 affects the expression of MMP12 mRNA and protein. If Egr1 is overexpressed, it will lead to an increase in the expression of MMP12, resulting in the decomposition of matrix proteins or the promotion of the expression of other MMPs, ultimately leading to the occurrence of IBD.

In diseases characterized by a high expression of Egr1, reducing the expression of Egr1 using small-molecule drugs or inhibitors may be a treatment choice. In a mouse model of prostate cancers, it was observed that the administration of antisense oligonucleotides could inhibit the expression of Egr1 and ameliorate tumor growth [36]. Regarding natural ingredients, curcumin has been shown to inhibit the activity of Egr1 in monocytes and colorectal cancer cells [37]. It can also inhibit the expression of Egr1 induced by IL-1β in lung epithelial cells, further reducing the expression of the inflammatory enzyme mPGES-1 [38]. In the clinical treatment of patients with IBD, infliximab, a humanized chimeric anti-TNF-α antibody, has demonstrated efficacy in relieving clinical symptoms by concurrently downregulating the expression of MMP1, MMP3, and MMP9 in these patients [39]. TNF-α induces EGR1 in various cell types, including the colon, and has been observed to mediate the TNF-stimulated expression of numerous genes [40,41,42]. We propose the presence of a positive regulatory loop involving proinflammatory cytokines and suggest that EGR1 may exacerbate the severity of IBD through a vicious cycle. Intervention to disrupt this cycle could offer new insights into practical strategies for managing IBD patients.

Additionally, our study found that both Egr1 gene KO mice and C57BL/6 wild-type mice experienced a decrease in their body weight after continuously receiving 0.5% DSS for 30 days. However, the degree of decrease in the wild-type mice was more dramatic. The signs of IBD were also evaluated from the feces of the mice, revealing more severe signs in the wild-type mice. This finding is worth exploring. On the 12th day, the wild-type mice began to show obvious signs, but their weight change did not decrease significantly until around the 20th day. We speculate that the average weight of a normal 8-week-old mouse is about 20–25 g, with a daily weight change of approximately 1.5 g. During the experiment, the mice consumed water and feed. If the mice did not eliminate the consumed water and food, and signs appeared during this period, the weight may not have decreased. Therefore, an analysis of signs may be more reliable than relying solely on the reference values of weight changes.

## 5. Conclusions

**Figure 6:**
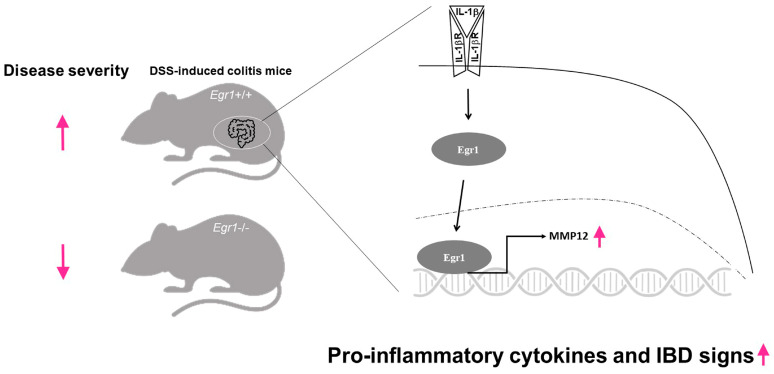
A schematic overview of the molecular mechanisms for regulating MMP12 in *Egr1* deficient mice. *Egr1* gene knockout (*Egr1*^−/−^) mice exhibit increased tolerance following exposure to DSS. In the intestines of wild-type mice (*Egr1*^+/+^), IL-1β facilitates the translocation of Egr1 into the cell nucleus, leading to the transcriptional activation of the MMP12 promoter and subsequent induction of MMP12 and other proinflammatory cytokines, such as IL-1β, IL-6, and TNF-α. Ultimately, these mice displayed more severe signs of IBD compared to *Egr1* gene knockout (*Egr1*^−/−^) mice.

The exact role of Egr1 in IBD remains elusive, yet our investigation sheds light on its impact. Our study reveals that Egr1 plays a pivotal role in modulating the expression of key proinflammatory cytokines, such as IL-1β, IL-6, and TNF-α. Moreover, we identified its interaction with the MMP12 promoter, thereby exerting regulatory control over MMP12 expression (Figure 6). These findings underscore the involvement of Egr1 in the inflammatory cascade, exerting influence on MMP expression. Consequently, dysregulated ECM cleavage due to heightened MMP activity exacerbates intestinal mucosal damage, thereby intensifying IBD symptoms. Looking ahead, elucidating the precise pathogenic pathways governed by Egr1 holds promise for refining IBD treatment strategies. By gaining deeper insights into Egr1’s mechanisms, we hope to devise more targeted and effective therapeutic interventions for IBD management.

## Data Availability

The data that support the findings of this study are available on request from the corresponding author.
